# Histological Subtypes Might Help Risk Stratification in Different Morphological Types of IPMNs: Back to the Future?

**DOI:** 10.3390/jcm13226759

**Published:** 2024-11-10

**Authors:** Giuseppe Anzillotti, Francesca Vespasiano, Chiara Maria Scandavini, Marco Del Chiaro, Asif Halimi, Alessandro Anselmo, Giuseppe Tisone, Carlos Fernández Moro, Zeeshan Ateeb, Urban Arnelo, J.-Matthias Löhr, Ernesto Sparrelid, Roberto Valente

**Affiliations:** 1 Department of Clinical Science, Intervention and Technology (CLINTEC), Karolinska Institute, 141 52 Stockholm, Sweden; doc.anzillotti@gmail.com (G.A.); vespasiano.francesca@gmail.com (F.V.); chiara.scanda@hotmail.it (C.M.S.); asif.halimi@regionvasterbotten.se (A.H.); zeeshan.ateeb@ki.se (Z.A.); urban.arnelo@regionvasterbotten.se (U.A.); matthias.lohr@ki.se (J.-M.L.); ernesto.sparrelid@ki.se (E.S.); 2 Department of Biomedical Sciences, Humanitas University, Via Rita Levi Montalcini 4, Pieve Emanuele, 20072 Milan, Italy; 3 IRCCS Humanitas Research Hospital, Via Manzoni 56, Rozzano, 20089 Milan, Italy; 4 Unit of Cardiac Sciences, Department of Medicine, Campus Bio-Medico University of Rome, 00128 Rome, Italy; 5 Department of Diagnostic and Interventions, Surgery, Umeå University, 901 87 Umeå, Sweden; 6 Division of Surgical Oncology, Department of Surgery, University of Colorado Anschutz Medical Campus, Denver, CO 80045, USA; marco.delchiaro@cuanschutz.edu; 7 Transplant Unit, University of Roma Tor Vergata, 00133 Rome, Italy; alessandroanselmo.ptv@gmail.com (A.A.); tisone@med.uniroma2.it (G.T.); 8 Department of Clinical Pathology/Cytology, Division of Pathology, Karolinska University Hospital, 141 57 Huddinge, Sweden; carlos.fernandez.moro@ki.se; 9 Department of Upper Digestive Diseases, Karolinska University Hospital, 141 57 Stockholm, Sweden

**Keywords:** pancreatic cancer, intraductal papillary mucinous neoplasm (IPMN), cyst, histology, main duct, branch duct, pancreatic ductal adenocarcinoma

## Abstract

**Background:** Intraductal papillary mucinous neoplasms (IPMNs) display four histological subtypes: gastric foveolar, pancreaticobiliary, intestinal, and oncocytic. All of these subtypes harbor a different risk of cancer development. The clinical impact of these subtypes concerning the occurrence of high-grade dysplasia (HGD)/cancer (C) in specific morphological types, such as branch-duct (BD), main-duct (MD), and mixed-type (MT) IPMNs, has been less investigated. Hence, our aim was to investigate the prevalence of histological subtypes and their possible association with HGD/C concerning morphologically different IPMNs. **Methods:** This was a retrospective review of demographics, risk factors, and histological features in a surgical cohort of patients having undergone resection for suspect malignant IPMNs at a high-volume tertiary center from 2007 to 2017. **Results:** A total of 273 patients were resected for IPMNs from during the study period, of which 188 were included in the final analysis. With sex- and age-adjusted multivariable logistic regression analysis across the entire cohort, gastric foveolar subtypes were associated with a reduced prevalence of HGD/C (OR = 0.30; 0.11–0.81, 95% CI, 95%CI; *p* = 0.01). With univariable logistic regression analysis, in the BD-IPMN subgroup, the pancreaticobiliary subtype was associated with an increased prevalence of HGD/C (OR = 18.50, 1.03–329.65, 95% CI; *p* = 0.04). In MD- and MT-IPMNs, the gastric foveolar subtype was associated with a decreased prevalence of HGD/cancer (OR = 0.30, 0.13–0.69, 95% CI; *p* = 0.004). **Conclusions:** In MD and MT-IPMNs, the gastric-foveolar subtype is associated with a lower prevalence of HGD/C, possibly identifying in such a high-risk group, a subgroup with more indolent behavior. In BD-IPMNs, the pancreaticobiliary subtype is associated with a higher prevalence of HGD/C, conversely identifying among those patients, a subgroup deserving special attention.

## 1. Introduction

Pancreatic cancer will be the second cause of cancer-related deaths by 2030. Therefore, it constitutes a medical emergency [1,2]. Intraductal papillary mucinous neoplasms (IPMNs) are increasingly considered to be the precursor lesions of this type of cancer. They harbor a variety of histological lesions that range from low-grade dysplasia (LGD) to cancer (C) [3,4,5]. Nevertheless, the incidence of pancreatic cancer is far lower than the prevalence of IPMNs. Therefore, not all IPMNs will eventually develop into cancer [6].

A significant issue in the general management of IPMNs is that preoperative diagnostics, including computer tomography (CT) and magnetic resonance imaging (MRI), suffer from low overall accuracy. Moreover, no available tests can predict the grade of preoperative dysplasia [7]. IPMNs are morphologically divided into three main types: branch-duct IPMNs (BD-IPMNs), main-duct IPMNs (MD-IPMNs), and mixed-type IPMNs (MT-IPMNs). MD-IPMNs and MT-IPMNs are characterized by segmental or diffuse dilatation of the main pancreatic duct with no cystic lesions [8]. Conversely, BD-IPMNs form clusters among the branch ducts, with a grape-like appearance, consisting in multiple lesions throughout the pancreas in up to 40% of cases [9]. The risk of malignancy is highly correlated to the morphological classification, being up to 38% for BD-IPMNs and up to 85% for MD-and MT-IPMNs [10]. Accordingly, given the higher risk of harboring high-grade dysplasia (HGD) and cancer in MD-IPMNs and MT-IPMNs, surgical resection should be warranted. On the other hand, the clear majority of BD-IPMNs can be safely monitored with cross-sectional imaging [11,12,13,14].

Histologically, IPMNs are further classified into four subtypes: pancreaticobiliary (PB), gastric foveolar (GF), oncocytic (O), and intestinal (I). The PB type consists in cells resembling cholangiopapillary neoplasms and with complex and thin-branching papillae. The GF type has cells resembling gastric foveolae, previously defined as the null-type IPMN. The O-type IPMN consists in cells with abundant, highly eosinophilic cytoplasm, forming thick papillae with intraepithelial lumina. Lastly, the I type resembles intestinal villous neoplasms with tall columnar epithelial cells [15]. All these subtypes are associated with a different overall risk of cancer [15]. The clinical implications of the co-existence of a specific histological subtype with the occurrence of HGD/C in specific morphological subtypes have been less extensively investigated.

As a matter of fact, morphological classification still represents the cornerstone of preoperative risk assessment and eventually guides clinical management. Moreover, considering the low preoperative diagnostic yield [7], there is an increasing need to sub-stratify patients within the same morphological subtype, identifying those who will eventually progress to develop cancer. Hence, the aim of the present study is to investigate the prevalence of histological subtypes (PB, GF, O, and I) in morphologically different types of IPMNs (MD-IPMNs, MT-IPMNs, and BD-IPMNs) and to evaluate their possible association with the findings of HGD/C in final surgical pathology specimens.

## 2. Materials and Methods

### 2.1. Study Design

This study was a retrospective, single-center cohort study. Consecutive patients had operations for suspected IPMNs between January 2007 and July 2017 at the Hepato-Pancreato-Biliary Unit in the Karolinska University Hospital in Stockholm, Sweden. This study received the approval of the local ethics committee (Stockholm EPN 2016/2542–31/1). We investigated demographics, radiological features, symptoms, and pathological features. We also investigated possible known risk-factors for PDACs, such as smoking and a new onset of diabetes. Recent-onset diabetes mellitus was defined as diabetes diagnosed in the latest 12 months before the diagnosis of IPMNs. Ever-smoker was considered to be a patient who had smoked for more than six months or more than 100 cigarettes in a lifetime [16]. The CA 19.9 was defined as increased if >37 U/mL [11,13].

All patients included had undergone preoperative MRI/MRCP or contrast-enhanced CT of the pancreas. We discussed all cases in the multidisciplinary conference before surgery [7]. The indication for surgical intervention was set according to the first edition of the International Association of Pancreatology (IAP) guidelines as at 2012 [17] and according to the European Experts Consensus from 2012 [18].

### 2.2. Pathological Assessment

Histological subtypes were classified morphologically. Further analysis of the immunohistochemical phenotype was defined according to the different mucin expression, which was previously defined as a discriminant to unequivocally identify the histological subtypes [15,19]. The main immunohistochemical markers were the following:

- The gastric foveolar type: expression of MUC5AC,
- The pancreaticobiliary type: expression of MUC1,
- The intestinal type: expression of MUC2, MUC5AC, and CDX2,
- Oncocytic type: expression of MUC5AC, MUC6, and sometimes HEPAR-1

When assessing the degree of epithelial dysplasia, according to the Baltimore consensus [20], only “low-grade” and “high-grade” dysplasia were considered.

Inclusion criterion was patients with a histologically confirmed IPMN.

Exclusion criteria were an unknown histological subtype and a “synchronous PDAC,” defined as a pancreatic ductal adenocarcinoma separated from the IPMN [21,22].

### 2.3. Statistical Analysis

We performed an initial descriptive analysis of the entire cohort to assess the prevalence of the different subtypes and the possible association with high-grade dysplasia or cancer. Subsequently, we sub-analyzed the groups of BD- and MD/MT-IPMNs.

The Chi-square or Fisher test were applied to analyze categorical variables, while Student’s *t*-test, Log-Rank, and Mann–Whitney were used with continuous variables as indicated. We further analyzed statistically significant results through sex- and age-adjusted univariable and multivariable logistic regression analysis. The statistical software was Med Calc v20.027(MarienKirke, Schaerbeek, Belgium).

## 3. Results

### 3.1. Study Population

**Figure 1:**
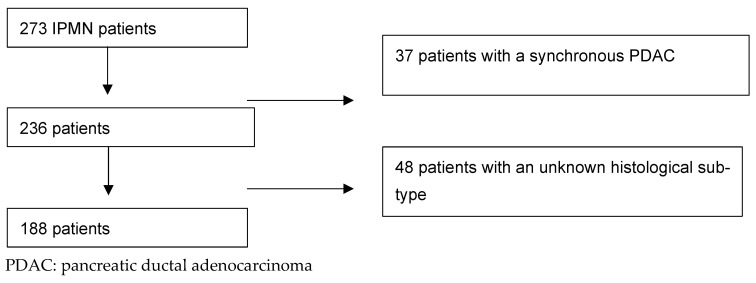
Inclusion flow-chart according to CONSORT.

Overall, 273 patients were operated for IPMNs between January 2007 and July 2017 at the HPB Unit in the Karolinska University Hospital, Stockholm. A total of 188 patients were included in the final analysis, and the mean age of the patients was 72.32 (67.71–76.93; 95% CI). Of these, 89 (47.34%) were men (Figure 1).

Table 1a,b shows an overview of demographics, clinical and radiological features, and histological characteristics.

Table 2 shows the association of several patients’ features and the degree of dysplasia.

### 3.2. Surgical Procedures

Eighty-three patients (44.14%) underwent pancreaticoduodenectomy, sixty-five (34.57%) distal pancreatectomy, and thirty-three (17.55%) total pancreatectomy. One patient (0.53%) received central pancreatectomy, five patients (2.65%) enucleation, and one patient (0.53%) atypical resection.

### 3.3. Clinical Characteristics

Jaundice was statistically associated with a higher prevalence of HGD/C compared to low-grade dysplasia (LGD) (21.3% vs. 2.1%, *p* < 0.0001). In univariable and multivariable sex- and age-adjusted logistic regression analysis, this represented a statistically significant risk factor (with an OR of 12.15, 2.74–53.81, 95% CI; *p* = 0.001 and an OR of 7.75, 1.52–39.31, 95% CI; *p* = 0.01). The incidental diagnosis of IPMNs was associated with a decreased prevalence of HGD/C at final histology (36.6% vs. 59.6%, *p* = 0.001). These data were confirmed at univariable logistic regression analysis: with an OR of 0.39 (0.21–0.72, 95% CI; *p* = 0.002). The median serum CA19-9 was significantly higher in patients displaying HGD (36 U/mL, 24–63, 95% CI) compared to LGD (9.45 U/mL, 7.52–13, 95%CI); *p* < 0.0001. When analyzed as a continuous variable, every unit increase of CA19.9 was confirmed to be associated with higher chances of finding HGD/C. Univariable and multivariable sex- and age-adjusted logistic regression analysis shown respectively an OR of 1.008; 1.002–1.01, 95% CI; *p* = 0.005 and an OR of 1.005; 1.0007–1.01, 95% CI; *p* = 0.02. On the other hand, symptoms such as abdominal pain and clinical manifestations such as acute pancreatitis were not associated with an increased risk of displaying HGD/C in the final specimen (*p* = 0.10 and *p* = 0.26, respectively).

### 3.4. Possible Risk Factors in the Overlap with PDACs

None of the potential risk factors considered, such as smoking, diabetes, and chronic pancreatitis, were significantly statistically associated with HGD/C.

### 3.5. Localization and Radiological Features

Multifocal disease and pancreatic head localization were statistically borderline in significance in association to HGD/C (respectively 39.4% vs. 53.2%, *p* = 0.05 and 34% vs. 21.3%, *p* = 0.05).

The mean caliber of the MPD was 7.4 mm (6.6–8.2; 95%CI). Patients with HGD/C displayed a statistically significant larger diameter of the MPD compared to patients harboring LGD: 8.8 mm (7.6–10) vs. 6 mm (5.1–6.9), *p* = 0.003. When considered as a continuous variable, every mm increase in the diameter of the MPD was associated with an increased risk of harboring HGD/C. Univariable sex- and age-adjusted logistic regression analysis has shown an OR of 1.13 (1.05–1.25), *p* = 0.0007.

The mean cystic diameter was not associated with a higher prevalence of HGD/C compared to LGD, (21.1 mm; 16.1–26.2, 95% CI vs. 26.2 mm; 22–30.5, 95% CI; *p* = 0.09). The presence of mural nodules was not associated with statistically significant differences in the rate of HGD/C and LGD, respectively (6.5% vs. 8.5%, *p* = 0.59).

### 3.6. Pathological Features

According to morphological classification, MD-IPMNs, MT-IPMNs, and BD-IPMNs were present in 5%, 78%, and 16% of the patients, respectively. LGD was present in 94 (50%) patients, HGD in 42 (22%), and IPMNs with invasive carcinomata in 52 patients (28%).

**Figure 2:**
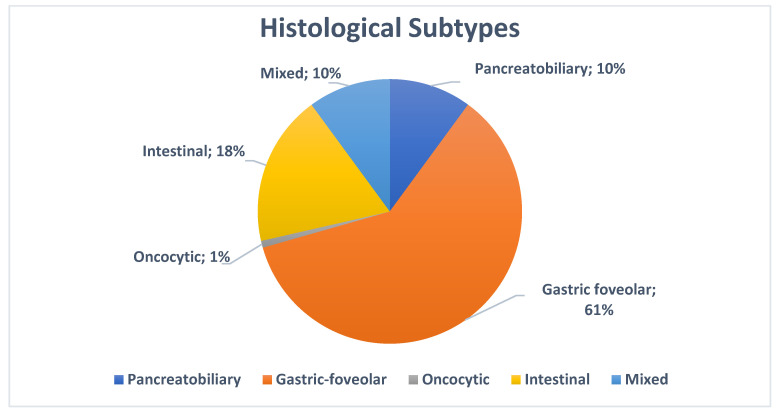
Prevalence of histological subtypes in the entire cohort (in %).

We then investigated the prevalence of histological subtypes in the entire cohort (Figure 2): of the cohort, 24 patients (13%) displayed the pancreaticobiliary subtype, 144 (77%) the gastric foveolar subtype, 44 (23%) the intestinal subtype, and 2 (1%) the oncocytic subtype. In 24 (13%), there were mixed phenotypes.

The gastric foveolar subtype was associated with less prevalence of HGD compared to LGD in the entire cohort (64% vs. 89%, *p* < 0.0001). Such an association was also confirmed by sex- and age-adjusted univariable and multivariable logistic regression analysis, with an OR of 0.21 (0.09–0.46, 95% CI; *p* = 0.0001) and an OR of 0.30 (0.11–0.81, 95% CI; *p* = 0.01). Conversely, the intestinal subtype was more likely to be associated with HGD/C rather than LGD (32% vs. 15%, *p* = 0.006). Such an association was also confirmed by sex- and age-adjusted univariable logistic regression analysis, with an OR of 2.54 (1.23–5.24, 95% CI; *p* = 0.01), as shown in Table 3a,b.

### 3.7. Sub-Analysis of BD-IPMNs

In BD-IPMNs the pancreaticobiliary subtype was significantly associated with a higher prevalence of HGD/C compared to LGD (33% vs. 4%, *p* = 0.03), Table 4a.

**Figure 3:**
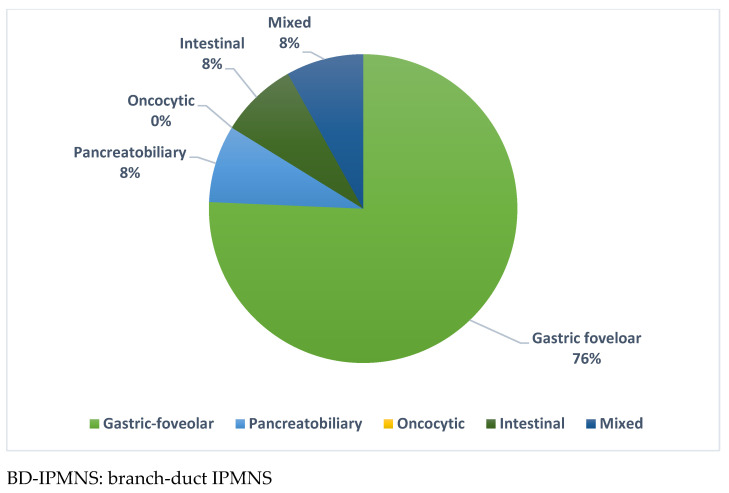
Prevalence of histological subtypes in BD-IPMNs.

Such an association was also confirmed by a univariable logistic regression analysis, with an OR of 18.50 (1.03–329.65, 95% CI, *p* = 0.04). Also, the intestinal subtype was associated with a higher prevalence of HGD/C (33% vs. 4%, *p* = 0.03), but a univariable logistic regression analysis did not reach any statistical significance, with an OR of 11.54 (0.63–210.67, 95%CI, *p* = 0.09). In contrast, the gastric foveolar subtype displayed a lower prevalence of HGD/C compared to LGD (50% vs. 100%, *p* = 0.0003). The prevalence of histological subtypes in the subgroup of BD-IPMNs is shown in Figure 3.

### 3.8. Sub-Analysis in MD/MT-IPMNs

In MD/MT-IPMNs, the gastric foveolar subtype was associated with a lower prevalence of HGD compared to LGD (65% vs. 86%, *p* = 0.003), Table 4b.

**Figure 4:**
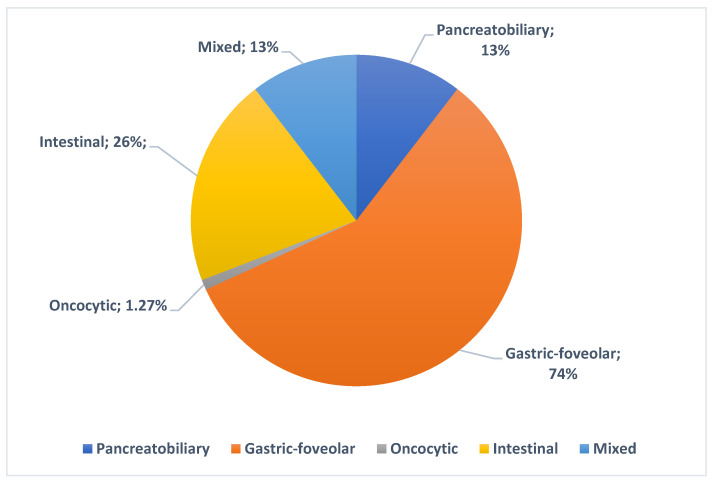
Prevalence of histological subtypes in MD/MT-IPMNs.

It displayed a lower risk of harboring HGD/C, including when performing a sex- and age-adjusted univariable logistic regression analysis, with OR = 0.30 (0.13–0.69, 95% CI; *p* = 0.004). Figure 4 shows an overview of the prevalence of histological subtypes in the subgroups of MD/MT-IPMNs.

## 4. Discussion

This study investigated the prevalence of different histological subtypes and their association with the finding of HGD/C in a surgical specimen. Consistent with previous studies, we found that the gastric foveolar subtype was the most prevalent and was less associated with HGD/C. We also found a decreasing trend in the prevalence of intestinal and pancreaticobiliary subtypes, both associated with an increased prevalence of HGD/C. Eventually, we confirmed the rarity and almost indolence of the oncocytic subtype.

This pattern of prevalence and association with HGD/C is nothing new and, once again, it is consistent with previous evidence [23,24]. The individual nature of our study can be seen in that we tried to correlate a body of evidence with different morphological IPMN subtypes (MD-IPMN, MT-IPMN, and BD-IPMN). We have hypothesized that BD-IPMNs and IPMNs involving the main-duct (MD-IPMNs and MT-IPMNs), harboring a different risk of cancer, might also have a different underlying prevalence of histological subtypes and are also associated with a different risk of cancer. In MD-IPMN and MT-IPMN patients, we found that the gastric foveolar subtype was associated with a decreased risk of presenting HGD/C. Conversely, in BD-IPMN patients, we found that the pancreaticobiliary subtype was associated with a higher prevalence of HGD/C.

In recent years, several studies investigated the possible role of histological subtypes in IPMNs, mostly focusing on prognostic relevance in terms of patients’ survival [15,25,26,27,28]. Koh et al. and Furukawa et al. both agreed on the influence of histological subtypes in the long-term prognosis of patients affected by IPMNs. On the other hand, another major study on the topic conducted by Kang et al. does not consider the histological subtype to be a relevant factor influencing the outcome. Referring to pathological features, Assay et al. showed that the most invasive IPMN pattern was the intestinal subtype that usually harbors colloidal carcinomata, with a better prognosis than conventional PDACs [29]. The pancreaticobiliary subtype, when invasive, showed a prognosis comparable to conventional PDACs [30]. The gastric foveolar subtype was less frequently associated with being invasive [31,32,33,34]. The oncocytic subtype has a unique phenotype and different biology. It harbors a better prognosis when associated with an invasive component [35,36]. Kang et al. also investigated the prognostic value of the histological subtype and its association with the grade of dysplasia. The gastric foveolar type was less frequently associated with HGD compared to other subtypes, but Kang et al. did not evaluate whether this relation was consistent in morphological IPMN subgroups. Furthermore, they did not investigate the possible coexistence of different histological subtypes, focusing only on the predominant component [28]. Recently, Rong et al. have described on a cohort of 121 patients the prevalence of different histological subtypes and the prevalence of dysplasia or invasive components [37]. As a point of difference, in the current study, we tried to investigate whether to display a specific histological subtype might have harbored a different risk for high-grade lesions (HGD/cancer) not only per se but also concerning a specific morphological subtype. Although much has been said of histological subtypes and the risk for cancer, we think that the debate is still open, even considering the continuous evolution of endoscopic diagnostic tools that might allow preoperative better sampling.

One of the distinctive aspects of the current study lies in the correlation between histological subtypes and morphology (MD-, MT-, and BD-IPMNs). Nowadays, the latter represents the main feature for considering surgery. On the other hand, it is becoming more and more evident how just a minority of IPMNs will eventually progress to cancer, and recent evidence even suggested slowing down and even discontinuing the follow-up of the so-called “trivial IPMNs” [38,39].

We believe that the current article attempts to look at an old issue from a new perspective. Our attempt is to approach the diagnostic and prognostic issue (low diagnostic accuracy at preoperative imaging and uncertainty regarding progression) considering histological subtype assessment as a weapon to enhance the risk stratification of such patients. Endoscopy-assisted procedures, such as pancreatoscopy and ultrasound, might help in characterizing histological subtypes and selecting the best candidates for surgery [40,41,42].

Furthermore, we investigated the presence of the eventual metachronous histological subtype. We could not find any specific association between mosaic histological subtypes with HGD/C. The current study displays some weaknesses. Firstly, the major limitation in histological characterization is the possible evolution from one subtype to another. For example, the pancreaticobiliary subtype may be considered a higher grade of the gastric foveolar subtype [43], and this could limit the impact of our findings. Secondly, we could have encountered a selection bias. In fact, the current study is based on a surgical selection of patients that may not reflect the characteristics of the entire population. Thirdly, the retrospective nature of our data and the lack of a preliminary power analysis may be another limitation. This study also has several strengths. The sole inclusion of patients who had received operations allowed us to analyze data from histologically confirmed IPMNs. This aspect is particularly noteworthy when considering that the preoperative diagnostic yield is woefully low. Therefore, most of the studies on non-operated IPMNs rely on presumed diagnoses [7].

In the last ten years, several guidelines [11,17,18,44,45,46,47,48] tried to reach conclusions on the optimal management of IPMNs. However, none of them has been able to provide accurate information on the assessment of the grade of preoperative dysplasia.

Our findings may imply opportunities for further improvements in the preoperative analysis, particularly tissue sampling through endoscopic modalities that often shed light on borderline and unclear situations [48].

Hopefully, the further development of new endoscopic tools (e.g., pancreatoscopic-guided sampling and EUS-guided histological characterization) will allow histological subtype assessments with an overall improvement of the preoperative diagnostic yield [40]. These improvements may result in the application of treatments tailored to the context of preventive pancreatic surgery programs.

Last but not least, this information might be useful in cancer risk stratification during pancreatic surgery. One of the main issues is finding LGD on the transection margin during pancreatic surgery. Although residual LGD in the MPD is technically a residual MD-IPMN, no convincing evidence supports the extension of the resection margin to avoid cancer reoccurring/redeveloping [11]. In this context, our findings might stratify this category of patients, according to the presence of a high- or low-risk histological subtype. In other words, finding LGD in a frozen section with an intestinal subtype might support a more aggressive strategy to avoid possible recurrences on the remnant pancreas. On the other hand, finding LGD in the presence of a gastric foveolar subtype, which is associated with a 70% decreased risk of cancer, might justify a more conservative approach. Finding BD-IPMNs with a pancreaticobiliary subtype might support a more aggressive strategy, even in the absence of other high-risk features. Of course, we cannot recommend a change in management policy based on our data. In the current study, we can only observe an association and not a causative effect. Furthermore, possibly multicentric longitudinal studies will be needed to confirm and validate our observations to turn them into a possible clinical application.

## 5. Conclusions

In this study, the gastric foveolar subtype, which is the most prevalent subtype, is associated with a lower prevalence of HGD/C, identifying a group of patients at low risk of progression to invasive cancer.

These data seem to be particularly true in MD- and MT-IPMNs, possibly identifying a possible subgroup with more indolent behavior for the first time in this high-risk group. In BD-IPMNs, the pancreaticobiliary subtype is associated with a higher prevalence of HGD/C, identifying, conversely, within a common and generally indolent group, a subgroup that might deserve special attention. Further studies are needed to confirm analogous associations and, therefore, to continue to shed light on this crucial challenging dilemma in pancreatic diseases.

## Figures and Tables

**Table 1:** (**a**): Clinical characteristics of 188 IPMN patients included in the analysis, n (%). (**b**): Histological characteristics of 188 IPMN patients included in the analysis, n (%).

(a)	
**Patients**	188
**Mean age**	72.3 (67.7–76.9; 95% CI)
**Sex (male)**	89 (47.3)
**Mean BMI**	25.9 (25.2–26.5; 95% CI)
**Smoking**	48 (25.5)
**Chronic Pancreatitis**	7 (3.7)
**Diabetes**	40 (21.2)
**Incidental Diagnosis**	90 (48.1)
**Abdominal Pain**	39 (20.7)
Weight Loss *	28 (14.8)
**Head localization**	52 (27.6)
**Median Ca19.9**	18 (14.9–21.0; 95% CI)
**Multifocal disease**	87 (46.2)
**Mean MPD diameter (mm)**	7.4 (6.6–8.2; 95% CI)
**Mean Cyst diameter (mm)**	23.71 (20.4–27.0; 95% CI)
**Pancreatoduodenectomy**	83 (44.1)
**Distal Pancreatectomy**	65 (34.57)
**Enucleation**	5 (2.65)
**Central pancreatectomy**	1 (0.53)
**Clinical and Radiological Indications for surgery**	**n (%)**
MPD ≥ 1 cm	42 (22.4)
Cyst diameter ≥ 40 (mm)	45 (23.9)
Mural nodules	14 (7.4)
MPD 5–9.9 mm	84 (44.6)
Recent Onset Diabetes (<1 yr)	2 (1.0)
Jaundice	22 (11.7)
Acute Pancreatitis	23 (12.2)
Elevated Ca19.9 (>37 U/mL)	56 (29.7)
(b)	
**Histological types and subtypes**	n (%)
**Total**	188 (100)
**MD IPMN**	10 (5.3)
**MT IPMN**	147 (78.1)
**BD IPMN**	31 (16.4)
**LGD**	94 (50)
**HGD**	42 (22.3)
**Invasive IPMN**	52 (27.6)
**Pancreaticobiliary type**	24 (12.7)
**Gastric foveolar type**	144 (76.5)
**Oncocytic type**	2 (1.0)
**Intestinal type**	44 (23.4)
**Mixed**	24 (12.7)
**Mixed PG**	8 (33.3)
**Mixed PO**	0 (0)
**Mixed PI**	3 (12.5)
**Mixed GO**	0 (0)
**Mixed GI**	17 (70.8)
**Mixed OI**	0 (0)
**Multiple**	2 (8.3)

MPD: Main Pancreatic Duct; BMI: body mass index * Weight loss was defined as an unintentional decrease in body weight (related neither to increased physical activity nor to the decrease of daily calorie intake). MD-IPMN: main-duct IPMN; MT-IPMN: mixed-type IPMN; BD-IPMN: branch-duct IPMN; LGD: low-grade dysplasia; HGD: high-grade dysplasia; PG: pancreaticobiliary-gastric foveolar; PO: pancreaticobiliary-oncocytic; PG: pancreaticobiliary-gastric foveolar; PI: pancreaticobiliary-intestinal; GO: gastric foveolar-oncocytic; GI: gastric foveolar-intestinal; OI: oncocytic-intestinal.

**Table 2:** Possible associations between patients’ characteristics and the presence of HGD/cancer n (%). Measures of associations were calculated with the chi-square for categorical variables, Mann–Whitney/Log-Rank, for non-normal distributed continuous variables, and Student’s *t*-test for continuous normal distributed variables.

Patients	HGD/Cancer	LGD/Benign	*p*-Value
**MEAN AGE**	73.0 (66.3–79.8; 95% CI)	71.5 (65.1–77.9; 95% CI)	0.5
**SEX (MALE)**	49 (52.1)	40 (42.6)	0.18
**MEDIAN BMI**	25.3 (24.5–26.2; 95% CI)	26.4 (25.5–27.4; 95% CI)	0.15
**SMOKING**	20 (21.3)	28 (29.8)	0.18
**DIABETES**	23 (24.5)	17 (18.1)	0.28
**INCIDENTAL DIAGNOSIS**	34 (36.6)	56 (59.6)	0.001
**JAUNDICE**	20 (21.3)	2 (2.1)	<0.0001
**ACUTE PANCREATITIS**	9 (9.6)	14 (14.9)	0.26
**ABDOMINAL PAIN**	24 (25.5)	15 (16.0)	0.10
**HEAD LOCALIZATION**	32 (34)	20 (21.3)	0.05
**MEDIAN CA19.9**	36 (24–63; 95% CI)	9.45 (7.5–13.0; 95% CI)	<0.0001
**MULTIFOCAL DISEASE**	37 (39.4)	50 (53.2)	0.05
**MEAN MPD DIAMETER (MM)**	8.8 (7.6–10.0; 95% CI)	6.07 (5.1–6.9; 95% CI)	0.003
**MEAN CYST DIAMETER (MM)**	21.1 (16.1–26.2; 95% CI)	26.27 (22.0–30.5; 5% CI)	0.09
**MURAL NODULES**	6 (6.5)	8 (8.5)	0.59
**PANCREATOBILIATY**	16 (17)	8 (8.5)	0.08
**GASTRIC FOVEOLAR**	60 (63.8)	84 (89.4)	<0.0001
**ONCOCITIC**	1 (1.1)	1 (1.1)	1
**INTESTINAL**	30 (31.9)	14 (14.9)	0.006
**MIXED**	11 (11.7)	13 (13.8)	0.66
**PG**	5 (5.3)	3 (3.2)	0.47
**PI**	2 (2.1)	1 (1.1)	0.56
**PO**	-	-	-
**GO**	-	-	-
**GI**	8 (8.5)	9 (9.6)	0.79
**OI**	-	-	-

MPD: Main Pancreatic Duct; LGD: low-grade dysplasia; HGD: high-grade dysplasia; PG: pancreaticobiliary-gastric foveolar; PO: pancreaticobiliary-oncocytic; PG: pancreaticobiliary-gastric foveolar; PI: pancreaticobiliary-intestinal; GO: gastric foveolar-oncocytic; GI: gastric foveolar-intestinal; OI: oncocytic-intestinal.

**Table 3:** (**a**): Sex- and age-adjusted univariable logistic regression analysis in the entire cohort. (**b**): Sex- and age-adjusted multivariable logistic regression analysis in the entire cohort.

**(a)**		
**Patients**	**Univariable OR (95%CI)**	***p*** **-Value**
Incidental diagnosis	0.39 (0.21–0.72)	0.002
Jaundice	12.15 (2.74–53.81)	0.001
Serum Ca19.9	1.008 (1.002–1.01)	0.005
MPD diameter (mm)	1.13 (1.05–1.25)	0.0007
Intestinal	2.54 (1.23–5.24)	0.01
Gastric foveolar	0.21 (0.09–0.46)	0.0001
**(b)**		
**Patients**	**OR Multivariable (95%CI)**	***p*** **-Value**
Incidental diagnosis	1.02 (0.48–2.16)	0.94
Jaundice	7.75 (1.52–39.31)	0.01
Serum Ca19.9	1.005 (1.0007–1.01)	0.02
MPD diameter (mm)	1.07 (0.99–1.15)	0.07
Intestinal	1.58 (0.60–4.20)	0.35
Gastric foveolar	0.30 (0.11–0.81)	0.01

MPD: Main Pancreatic Duct.

**Table 4:** (**a**): Sub-analysis of a possible association between histological subtypes and the presence of HGD/cancer in BD-IPMNs. (**b**): Possible associations between histological subtypes and HGD/C in MD/MT-IPMNs.

**(a)**			
**Patients**	**HGD/Cancer**	**LGD/Benign**	***p*** **-Value**
Pancreaticobiliary	2 (33.3%)	1 (4%)	0.03
Gastric foveolar	3 (50%)	25 (100%)	0.0003
Oncocytic	-	-	-
Intestinal	2 (33.30%)	1 (4.0%)	0.03
**(b)**			
**Patients**	**HGD/Cancer**	**LGD/Benign**	***p*** **-Value**
Pancreaticobiliary	14 (15.9%)	7 (10.1%)	0.29
Gastric foveolar	57 (64.8%)	59 (85.5%)	0.003
Oncocytic	1 (1.1%)	1 (1.4%)	0.86
Intestinal	28 (31.8%)	13 (18.8%)	0.06

HGD: high-grade dysplasia; LGD: low-grade dysplasia; BD-IPMN: branch-duct IPMN. MD/MT-IPMN: main-duct/mixed-type IPMN.

## Data Availability

All the retrieved results are presented in the published paper. Raw data can be asked to the corresponding author, upon reasonable request.
